# Ketoprofen Combined with UVA Irradiation Exerts Higher Selectivity in the Mode of Action against Melanotic Melanoma Cells than against Normal Human Melanocytes

**DOI:** 10.3390/ijms222111966

**Published:** 2021-11-04

**Authors:** Klaudia Banach, Justyna Kowalska, Zuzanna Rzepka, Artur Beberok, Jakub Rok, Dorota Wrześniok

**Affiliations:** Department of Pharmaceutical Chemistry, Faculty of Pharmaceutical Sciences in Sosnowiec, Medical University of Silesia, 40-055 Katowice, Poland; kbanach@sum.edu.pl (K.B.); jkowalska@sum.edu.pl (J.K.); zrzepka@sum.edu.pl (Z.R.); abeberok@sum.edu.pl (A.B.); jrok@sum.edu.pl (J.R.)

**Keywords:** melanotic melanoma, UVA radiation, ketoprofen, melanocytes, COLO829

## Abstract

Malignant melanoma is responsible for the majority of skin cancer-related deaths. The methods of cancer treatment include surgical removal, chemotherapy, immunotherapy, and targeted therapy. However, neither of these methods gives satisfactory results. Therefore, the development of new anticancer therapeutic strategies is very important and may extend the life span of people suffering from melanoma. The aim of this study was to examine the effect of ketoprofen (KTP) and UVA radiation (UVAR) therapy on cell proliferation, apoptosis, and cell cycle distribution in both melanotic melanoma cells (COLO829) and human melanocytes (HEMn-DP) in relation to its supportive effect in the treatment of melanoma. The therapy combining the use of pre-incubation with KTP and UVAR causes a significant increase in the anti-proliferative properties of ketoprofen towards melanoma cells and the co-exposure of melanotic melanoma cells induced apoptosis shown as the mitochondrial membrane breakdown, cell-cycle deregulation, and DNA fragmentation. Moreover, co-treatment led to GSH depletion showing its pro-apoptotic effect dependent on ROS overproduction. The treatment did not show a significant effect on normal cells—melanocytes—which indicates its high selectivity. The results suggest a possible benefit from the use of the ketoprofen and ultraviolet A irradiation as a new concept of melanotic melanoma therapy.

## 1. Introduction

Malignant melanoma (*malignum melanoma*) is a tumor that originates from melanotic cells derived from neuroectodermal cells [1]. The reason for its neoplastic process is the transformation of melanocytes located in the skin, choroid, or mucosa. Most cases of de novo melanoma derive from epidermal melanocytes and it causes up to 80% of deaths in the patient suffering from skin cancer [2]. Melanoma is a type of skin cancer characterised by the high mortality rate, and unsuccessful therapy, which does not give satisfactory effects; in the 4th stage of clinical advancement, therapy does not give sufficient effects—the average life expectancy is 6–10 months, and less than 10% of patients survive 5 years [3,4,5]. Despite the beneficial reaction after the use of tyrosinase kinase inhibitors (e.g., dabrafenib, vemurafenib) or immune checkpoint inhibitors (e.g., ipilimumab, nivolumab), these new drugs have failed to provide adequate therapeutic effects in terms of stopping disease progression and considerably extending the life of patients with advanced metastases [6,7].

The photosensitive drug is activated upon exposition to UVAR, causing massive DNA strand breaks in cancer cells caused by reactive-oxygen species (ROS) [8]. Cancer cells are sensitive to an increase in ROS levels because they are characterised by basically high levels of ROS [9]. The increase in ROS in photosensitization may be helpful not only for evaluating phototoxicity to avoid drug-induced side effects, but also for using photosensitization as part of a cancer treatment variant [8]. The increase in ROS levels may induce a therapeutically beneficial pro-apoptotic effect that can be used to support the treatment of neoplastic diseases, including malignant melanoma. Long-term oxidative stress in cancer cells caused by ROS-generating drugs or therapies may exhaust their antioxidant mechanisms, which, after exceeding the appropriate concentration of free radicals in cells, may lead to apoptosis. Therefore, anti-cancer drugs may have an effect by increasing oxidative stress or reducing the ability of cells to defend antioxidants. The production of large amounts of ROS leads to irreversible damage and the depletion of antioxidant capacity and defense mechanisms of cancer cells. Moreover, one of the therapeutic methods assumes the reduction of the intracellular GSH concentration, which may cause a pro-apoptotic effect in tumor cells [9,10,11,12]. Due to the lack of efficacious methods of melanoma therapy, a new method of medical care for patients diagnosed with this type of tumor is still needed. Therefore, phototherapy can be deliberated as one of the new melanoma treatment.

Phototoxic drug reactions will presumably occur in all individuals and is exhibited upon receiving high enough doses of either the drug or the radiation at the proper wavelengths. In the phototoxic dermatitis, the photosensitizing agent, once triggered by UVR, straightway damages the tissue. One of the drugs with phototoxic properties is ketoprofen (KTP), classified as a nonsteroidal anti-inflammatory drug that is widely used in the pharmaceutical field, mainly because of its anti-inflammatory, analgesic, and antipyretic properties [13,14]. Topical ketoprofen is used to cure the pain and inflammation illnesses such as tendonitis, small joint osteoarthritis, back pain, phlebitis, and acute minor trauma (sprains, bruising) [15,16]. Moreover, the phototoxic effect of ketoprofen has been demonstrated in relation to breast cancer cells, where co-therapy using ketoprofen and UVAR caused a meaningful reduction of cell viability [17].

According to our earlier study, therapy combined with KTP and UVAR, exerted cytotoxic, anti-proliferative, and pro-apoptotic effects towards amelanotic melanoma cells (C32 line). The results of the study indicate the possible benefits of KTP and UVAR therapy in supporting the treatment of melanoma; therefore, we decided to assess these properties on a different melanoma cell line. So far, the effect of KTP and UVAR in melanotic melanoma (COLO829 line) has not been studied. In addition, the study on the normal cells—melanocytes (HEMn-DP)—was also expanded. This study was carried out as a continuation of the multistage research concerning the affinity of KTP and UVAR for cell proliferation, their cytotoxic and pro-apoptotic properties, and their impact on cell cycle in melanotic melanoma and melanocytes in relation to the anti-tumor effects to KTP and UVAR [18].

The aim of this study was to examine the influence of ketoprofen (KTP) and UVA radiation (UVAR) on cell proliferation, apoptosis, and cell cycle distribution in COLO829 melanoma cells in relation to their supportive effect in the treatment of melanoma. To assess the selectivity of ketoprofen combined with UVA irradiation mode of action, the experimental model was enriched by the use of human melanocytes.

## 2. Results

### 2.1. Effect on Cell Proliferation

**Figure 1:**
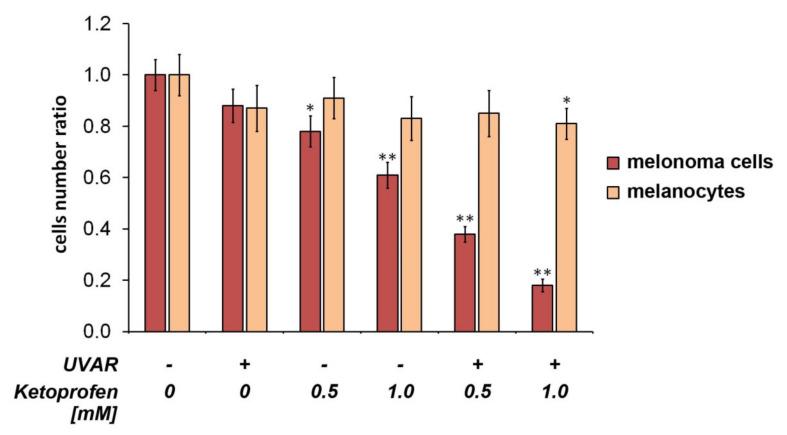
Impact of KTP and UVAR on the growth of COLO829 human melanotic melanoma cells and human melanocytes. The cells were treated with KTP at concentrations of 0.5 mM and 1.0 mM and then exposed to UVAR. The results are presented as the cell number ratio. * *p* < 0.05, ** *p* < 0.001 vs. control (one-way ANOVA, two-way ANOVA, Tukey’s test).

Data from the cell count assay indicated (Figure 1) that there was a decrease in the population of COLO829 melanoma cells exposed to KTP at a concentration of 0.5 mM and 1.0 mM, with the cell number ratio established as 0.78 and 0.68, respectively. We observed the greatest anti-proliferative effect towards melanoma cells after pre-incubation of cells with KTP at a concentration of 0.5 mM and 1.0 mM and irradiation with UVAR, where the ratio was 0.38 and 0.18 (Figure 1). It proves that the drug itself inhibits cell proliferation, but the use of the drug in combination with UVAR significantly potentiates this effect.

In contrast to COLO829 melanoma cells, a significant reduction in the number of HEMn-DP cells was noted only for cells pre-treated with KTP at a concentration of 1.0 mM and irradiated with UVAR (cell number ratio equal 0.81), showing KTP and UVAR combination as a selective in the mode of action against melanotic melanoma cells.

### 2.2. Co-Treatment with KTP and UVAR Induces Apoptosis in Melanoma Cells, but Not in Human Melanocytes

**Figure 2:**
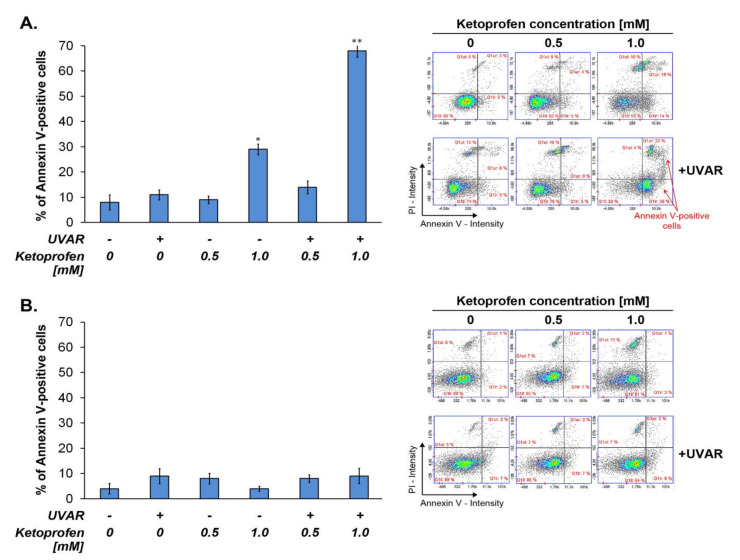
The annexin V/propidium iodide assays of (**A**) human melanoma cells COLO829 and (**B**) human melanocytes HEMn-DP exposed to KTP and UVAR are presented. Bar graphic represents mean values of the percentage of apoptotic cells and corresponding SD. * *p* < 0.05; ** *p* < 0.001 vs. control (one-way ANOVA, two-way ANOVA, Tukey’s test).

Annexin V/propidium iodide assay allows to distinguish cells between healthy and apoptotic. In the early stage of apoptosis, membrane-bound phosphatidylserine is transferred from the cytosol side to the outside of the cell. PS is joined by fluorescently labeled annexin V. To quantitatively measure apoptosis, COLO829 and HEMn-DP cells were treated with KTP (0.5 mM and 1.0 mM) and UVAR. The obtained results (Figure 2) showed that the drug (without UVAR) at the concentration of 1.0 mM increased the percentage of apoptotic cells by about 20%, when compared to the control. In turn, the percentage of apoptotic melanoma cells pre-incubated with KTP at the concentration of 1.0 mM and irradiated with UVAR increased by about 60%, in comparison to the control. Interestingly, in the case of melanocytes, KTP (without UVAR), UVAR alone, and KTP in combination with UVA did not affect changes in the number of healthy, early, and late apoptotic cells (Figure 2).

### 2.3. KTP in Combination with UVAR Decreases Mitochondrial Membrane Potential of Melanoma Cells

**Figure 3:**
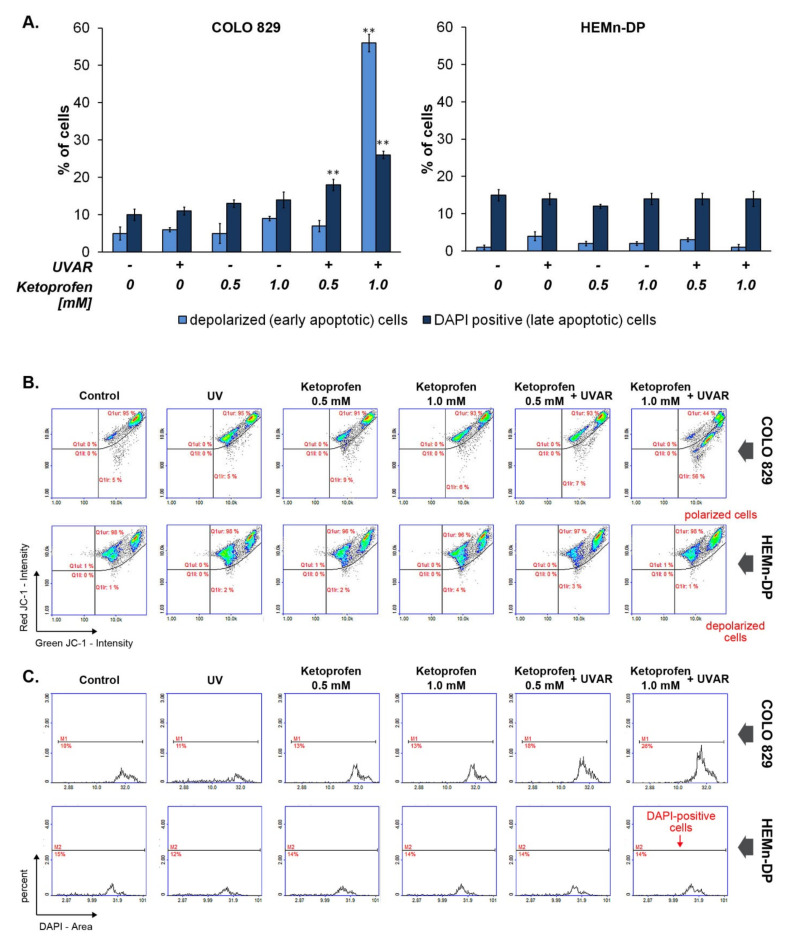
The mitochondrial transmembrane potential in melanotic COLO829 melanoma cells and human melanocytes HEMn-DP after co-treatment with KTP and UVAR. (**A**) Bar graphic representing changes in the proportion of cells with depolarized mitochondria and late apoptotic cells. (**B**) Scatter plots demonstrating changes in JC-1 intensity in tested cells. (**C**) Scatter plots showing changes in DAPI intensity in the studied cells. Mean values ± SD from three independent experiments performed in triplicate (*n* = 9) are presented. ** *p* < 0.001 vs. control (one-way ANOVA, two-way ANOVA, Tukey’s test).

The exposure of melanotic melanoma cells to KTP at the concentration of 1.0 mM and UVAR increased the percentage of mitochondrial membrane depolarized cells (early apoptotic cells) from 5 to 56%. The percentages of DAPI positive cells (late apoptotic cells) were about 18% and 26%, respectively, for samples: ketoprofen 0.5 mM + UVAR and ketoprofen 1.0 + UVAR, while the population of these cells in the control was determined to be about 10%. In turn, we did not notice any statistically significant changes in the number of polarized, depolarized, and DAPI positive melanocytes (Figure 3).

### 2.4. The Effect of KTP and UVAR on Melanoma and Melanocytes Cycle

**Figure 4:**
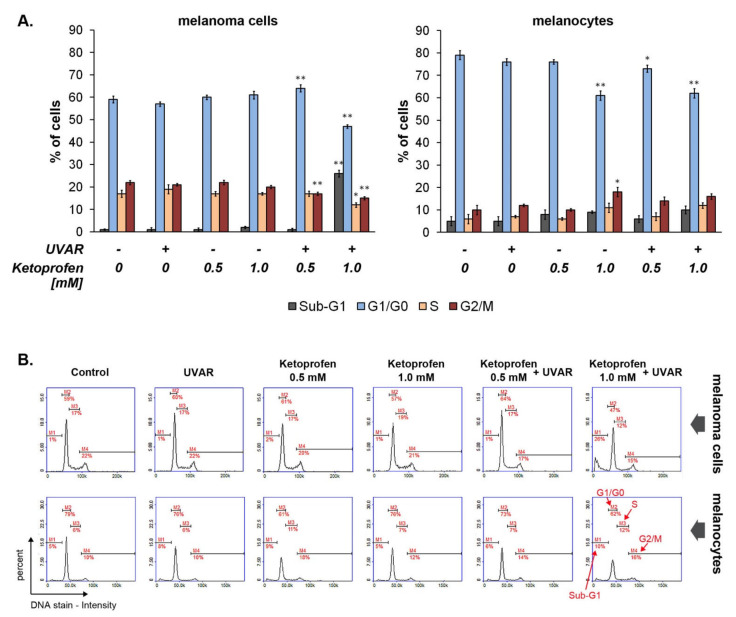
Effect of KTP and UVAR on cell cycle in melanoma cells and human melanocytes. (**A**) Bar graphic representing mean values ± SD of three independent experiments performed in at least three repetitions; * *p* < 0.05, ** *p* < 0.001 vs. control. (**B**) Representative histograms showing the cell cycle distribution (one-way ANOVA, two-way ANOVA, Tukey’s test).

The data presented in Figure 4 shows that the drug used at the highest concentration (1.0 mM) in combination with UVAR caused an increase in the number of COLO829 cells in the sub-G1 phase (by about 25% relative to the control). In turn, exposure of human melanocytes to drug and UVAR resulted in a weak–modulatory effect on the cell-cycle distribution with the rise of the percentage of cells in sub-G1 phase by about 5% when compared with the control.

### 2.5. The Influence of KTP and UVA Irradiation on DNA Fragmentation in Melanoma Cells and Melanocytes

**Figure 5:**
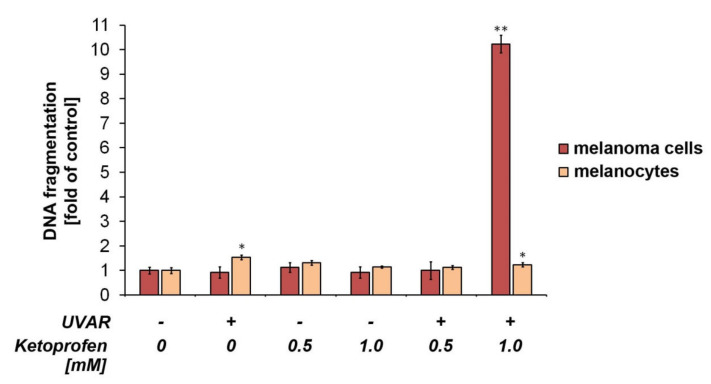
The impact of KTP and UVAR on DNA fragmentation in melanoma cells and melanocytes. Mean values of the percentage of cells with fragmented DNA and responding SD are shown as the bar graphic. * *p* < 0.05, ** *p* < 0.001 vs control (one-way ANOVA, two-way ANOVA, Tukey’s test).

The effect of KTP and/or UVAR on the DNA fragmentation was determined using image cytometry technique (Figure 5). The DNA fragmentation in COLO829 cells was detected after 24 h since irradiation and pre-incubation with ketoprofen at a concentration of 1.0 mM. The percentage of cells with fragmented DNA was found to be about 44%, while the value established for the control was about 4%. There was no increase in DNA fragmentation in the rest of the COLO829 cell samples. As shown in Figure 5, induction of DNA fragmentation with low intensity was detected in human melanocytes after UVA exposure (without drug) and pre-incubation with KTP at the concentration of 1.0 mM combined with UVAR where the percentage of cells with fragmented DNA increased by about 6% and 7%, respectively, compared with the control.

### 2.6. Co-Therapy with KTP and UVAR Decreases the Intracellular Level of Reduced Thiols

**Figure 6:**
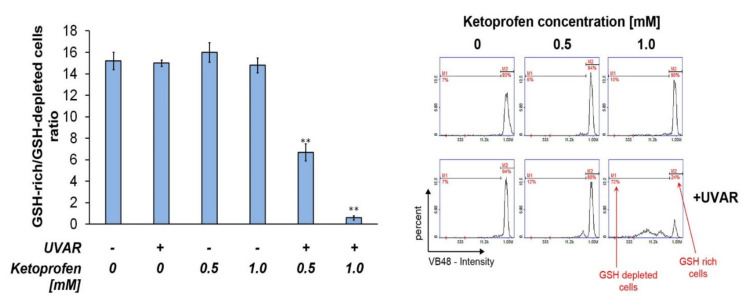
The co-treatment with KTP and UVAR affects the intracellular reduced thiols level in human melanotic melanoma cells. Representative scatter plots display distribution of the cell population for each tested group. Mean values ± SD of the percentage of cells with high and low level of reduced thiols are shown in the bar graphic. ** *p* < 0.001 vs. control (one-way ANOVA, two-way ANOVA, Tukey’s test).

In the current study, the impact of KTP and/or UVAR on the cellular GSH level was determined using fluorescence image microscopy. The obtained results indicated that simultaneous exposure to UVAR and KTP at both used concentrations (0.5 mM and 1.0 mM) decreased the cellular level of glutathione in its reduced state (Figure 6). After pre-treatment of COLO829 cells with KTP at the concentrations of 0.5 mM and 1.0 mM and irradiation with UVAR, the ratio of GSH rich/GSH depleted cells decreased by approximately two times and fifteen times, respectively, as compared with the controls.

### 2.7. Redox Homeostasis of Melanotic Melanoma Exposed to KTP and UVAR

**Figure 7:**
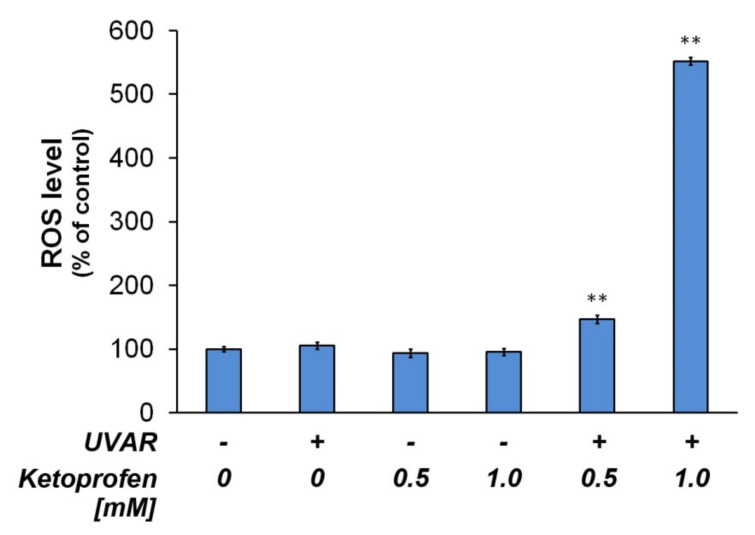
Redox homeostasis in melanoma COLO829 cell line exposed to KTP and UVAR. The cells were exposed to the drug (at concentrations of 0.5 and 1.0 mM for 24 h) and UVAR. Intracellular reactive oxygen species (ROS) level determined by H_2_DCFDA assay and calculated as the percentage of control. ** *p* < 0.001 vs. control (one-way ANOVA, two-way ANOVA, Tukey test).

To determine whether KTP and UVAR may affect redox homeostasis in melanotic melanoma cells, we evaluated the intracellular ROS level in COLO829 cells treated with KTP (0.5 mM and 1.0 mM) and UVAR. The assay with the H_2_DCFDA probe revealed an increase in ROS production in COLO829 cells exposed to KTP (at both concentrations—0.5 mM and 1.0 mM) combined with UVAR by 25% and 440%, respectively, compared to the control (Figure 7).

## 3. Discussion

Malignant melanoma is the most rapidly increasing cancer. The tumor arising from a melanocyte continues to carry the potential to be a deadly disease [2,3]. Despite various available methods of melanoma treatment, the results of therapy are not satisfying, mainly due to a lack of efficacy. All these issues generate the need of searching for new drugs and new methods of support treatment.

In current study, the anti-proliferative effect of KTP and UVAR was assessed by the evaluation of the cell number ratio. The drug and UVAR significantly reduced the growth of melanoma cells. KTP (0.5 mM and 1.0 mM) and UVAR reduced the cell ratio of melanoma cells to ca. 0.38 and 0.18, respectively (Figure 1). Additionally, the observations indicate a greater cytotoxic effect of KTP and UVAR on melanoma cells than on melanocytes.

In our previous study, a significant decrease in the number of COLO829 cells was also demonstrated after treatment with sulindac (without UVAR), which also belongs to the NSAIDs group [19].

One of the therapeutic methods which uses light radiation is PUVA (Psoralen Ultra-Violet A). PUVA phototherapy is applied to treat inflammatory skin diseases, including psoriasis, vitiligo, atopic dermatitis, contact dermatitis, cutaneous T-cell lymphoma, chronic pruritus, mastocytosis, or polymorphous light eruption. Psoralens are phototoxic compounds that, after absorbing photons of light, induce photochemical reactions that alter cellular homeostasis [20,21,22,23,24]. The cytotoxic effect on malignant melanoma cells has already been demonstrated in the case of phototherapy using psoralens (8-MOP, 5-MOP) and UVAR [25]. These observations confirm the results obtained in the current work which demonstrated the high cytotoxic effect of KTP combined with UVAR. Thus, phototherapy can be considered as one of the new methods of melanoma treatment.

In an attempt to explain the potential mechanism responsible for the observed reduction of the number of COLO829 cells after the tested conditions, it was noticed that KTP upon UVAR may cause phototoxic effects characteristic of its parent benzophenone compound, such as transfer of triplet–triplet energy to DNA bases, production of singlet oxygen, or the formation of reactive radicals species which could affect various biological structures [17].

Apoptosis is the programmed cell death characterised by a series of morphological changes, including membrane vesicle and cell shrinkage [26]. The hallmarks of apoptosis include phosphatidylserine (PS) externalization. PS regulates intercellular apoptosis by binding to proteins such as annexin V and lactadherin [27].

In reference to the obtained results, the influence of KTP and UVAR on the apoptosis process in melanotic melanoma cells was examined. The analysis of apoptosis involved the measurements of mitochondrial potential and annexin V.

According to the study on the COLO829 cell line, KTP (1.0 mM) and UVAR induced apoptosis. The percentage of melanoma apoptotic cells was about 70% (Figure 2). The results show and confirm relatively quickly and clearly a noticeable induction of apoptosis in investigated melanoma cells by KTP and UVAR. Moreover, no induction of apoptosis was observed in the melanocytes exposed to KTP and UVAR, indicating the selectivity of the method.

Similar effects were demonstrated after treatment with KTP and UVAR on amelanotic melanoma cells of the C32 line and the co-therapy caused an increase in the number of apoptotic cells [18]. Studies concerning the phototoxic effect of tetracycline and oxytetracycline on melanocytes showed significant cytotoxicity of the tested drugs, when compared with the data from the current study. Tetracyclines exhibited greater cytotoxic activity towards normal cells than KTP or KTP combined with UVAR [28,29]. Our results reveal that ketoprofen co-treatment with UVAR exerts a high selectivity in the targeted mode of action.

The intrinsic mitochondrial apoptotic pathway is characterised by alterations in the permeability of mitochondrial membranes [30]. In the present study, we used JC-1 staining. This fluorescent cationic dye manifests a potential-dependent accumulation in the mitochondria. In healthy cells, it accumulates in the mitochondria and forms dimers emitting red fluorescence. Furthermore, in apoptotic cells, JC-1 exists in the cytoplasm as a monomer exhibiting green fluorescence. As disruption of mitochondrial function, including changes in the mitochondrial transmembrane potential, represents an early event of apoptosis, the effect of KTP and UVAR on this parameter was evaluated.

To investigate whether the demonstrated induction of melanotic melanoma cells death was mediated by the mitochondrial pathway, a decline in mitochondrial membrane potential in COLO829 cells was analysed following KTP and UVAR treatment. In addition, the selectivity of the method was re-tested by assessing the mitochondrial potential in melanocytes. It was shown that the KTP concentration of 1.0 mM and UVAR induce mitochondrial membrane breakdown in melanoma cells (the percentages of depolarized cells reached 56%)—Figure 3. These results suggest that KTP and UVAR induce apoptosis via the mitochondria-mediated signaling pathway.

Similar conclusions were made in the study assessing the effect of KTP and UVAR treatment on amelanotic melanoma cells. The percentage of cells with depolarized mitochondrial membrane increased from 8 to 50% when C32 cells were exposed to KTP at a concentration of 1.0 mM and UVAR after a 48-h post-incubation time [18].

Rok et al. (2020) also analysed mitochondria involvement in the pro-apoptotic action of doxycycline towards melanoma cells. The revealed data showed that tetracycline antibiotics influence mitochondrial function. Exposure of cells to doxycycline caused a rise in the number of cells with depolarized mitochondrial membrane [9]. Similar changes were observed in our study, which indicates that the mechanism of apoptosis induced by KTP and UVAR in COLO829 melanoma cells involves mitochondria-dependent (intrinsic) pathways.

The cell cycle is a process composed of four phases: cell growth, DNA replication, separation of the duplicated chromosomes into daughter cells, and cell division. Additionally, the cell cycle has two basic parts: mitosis (M-phase) and interphase (G1, S, G2, G0-phase). M-phase includes karyokinesis and cytokinesis, respectively division of the nucleus and the cytoplasm. New cell cycle interphase, occurring after the M phase, prepares the cell for the next division [31]. Abnormalities of the cell cycle and cell division play a crucial role in tumorigenesis [32]. Cancer is considered as a disorder of the cell cycle, because it manifests by unregulated proliferation of cells [33]. Changes in the cell cycle can be caused by ROS overproduction. Oxidative stress may limit cell proliferation by inhibiting DNA synthesis, cell transition from G*0* to G*1*, or arresting in cycle checkpoints [34].

To determine the possible signaling pathways underlying the cytotoxic activity of KTP and UVAR, the analysis of the cell cycle of COLO829 melanotic melanoma cells and human melanocytes was made using a fluorescence image cytometer. In the performed analyses, the cells were distributed among four phases of the cell cycle: sub-G*1* (less than one DNA equivalent), G*1*/G*0* phase (one DNA equivalent), S-phase (DNA synthesis, in constant amount of DNA—represents cells in late apoptotic phase), and G*2*/M phase (double amount of DNA, prior to cell division) [35].

We have also demonstrated that KTP (1.0 mM) and UVAR cause sub-G1phase arrest in COLO829 cells (Figure 4). These findings are consistent with results obtained from the viability assay as well as analysis of apoptosis. The main limitation of therapeutic methods is their non-specific cytotoxicity, which damages cancer cells and also healthy cells. The demonstrated low cytotoxic effect of KTP and UVAR towards melanocytes suggests the selectivity of the research model.

Similar conclusions were drawn in the study assessing the effect of ciprofloxacin treatment on melanoma cells of the COLO829 line. Ciprofloxacin caused an increase in the number of cells in the sub-G*1* phase with simultaneous induction of DNA fragmentation [36].

The DNA fragmentation is the process occurring during the late stage of apoptosis. Activated factors responsible for DNA fragmentation (e.g., caspase-activated endonuclease), hydrolyze DNA into oligonucleosome-sized pieces, promoting chromatin packing. Double-stranded oligonucleosomal DNA fragments (also known as the DNA ladder) are considered as features of the apoptotic cell death [37,38,39].

The DNA damage in COLO829 cells was detected after pre-incubation with ketoprofen at the concentration of 1.0 mM and irradiation. The percentage of cells with fragmented DNA increased tenfold compared with control. As shown in Figure 5, induction of DNA fragmentation with low intensity was detected in human melanocytes after KTP (1.0 mM) and UVAR exposure where the percentage of cells with fragmented DNA increased by about 7% compared with the control (Figure 5).

Many in vitro studies of potential anti-melanoma therapies have shown an increase in the amount of fragmented DNA [5,9,36]. Similar conclusions were made in the study assessing the effect of KTP and UVAR treatment of C32 cell lines. KTP combined with UVAR induced DNA fragmentation, only 48 h after the irradiation procedure [18]. It was reported that exposure to ciprofloxacin at the concentration of 1.0 mM for 48 h and 72 h caused DNA fragmentation in melanoma cells [36]. The similar effects were demonstrated after treatment with MIM-1 alone and with a mixture of dacarbazine and MIM-1. It was observed an increase in the number of COLO829 cells with fragmented DNA [5]. The results obtained in another study indicate that doxycycline increases the number of melanoma cells with fragmented DNA [9].

Reduced glutathione (GSH) is a thiol tripeptide, which is composed of L-glutamate, cysteine, and glycine [40]. GSH is found mainly in the cytosol and a smaller amount in organelles such as the mitochondria, nucleus, and endoplasmic reticulum [41]. GSH takes part in many cellular processes, such as the neutralization of reactive oxygen species (ROS), DNA or protein synthesis, and signal transduction [42,43]. Moreover, GSH is the most frequent cellular antioxidant, which influences the regulation of many processes connected with tumor progression and sensitivity to cancer therapy [44].

One of the potential strategy of cancer treatment aims to modify redox signaling pathways in cancer cells by blocking the production of antioxidants, including GSH. It results in GSH depletion that increases the susceptibility of cancer cells to various forms of programmed cell death and sensitivity to chemotherapies [41]. As confirmation, many studies on mouse models of spontaneous oncogenesis have shown that inhibition of GSH synthesis could prevent tumor initiation [45]. In addition, cancer cells possess higher levels of ROS and a greatly powerful antioxidant system to avoid oxidative-stress-induced damage. In some cases, GSH depletion and high levels of ROS can trigger cell death [42,44]. Moreover, destruction of mitochondrial integrity, release of cytochrome c, and activation of caspases occurring during early stages of apoptosis was connected with the reduction of intracellular GSH [41,46].

After treatment of COLO829 cells with KTP at concentrations of 0.5 mM and 1.0 mM and irradiation with UVAR, the ratio of GSH rich/GSH depleted cells decreased two times and fifteen times, respectively, as compared with the controls (Figure 6).

Similar results were obtained in the study assessing the effect of KTP and UVAR treatment on amelanotic melanoma cells. The cytometric analysis revealed that the treatment of C32 cells with the KTP concentration (1.0 mM) and UVAR caused an increase in the percentage of cells with low GSH level by over 50% as compared with the controls. In addition, selectivity was tested in the mode of action of KTP and UVAR, and melanocytes were used as an experimental in vitro model. The obtained results indicate that KTP and UVAR had no effect on GSH levels in melanocytes [18]. The reason for the difference in GSH levels in melanoma and melanocytes is the different susceptibility of cells to oxidative-reduction imbalance. Neoplastic cells basically have an increased level of ROS compared to normal cells as a result of an imbalance between oxidants and antioxidants. Therefore, cancer cells are more sensitive to increasing ROS levels, which may inhibit the viability of melanoma cells and tumor growth [42]. These results suggest that KTP and UVAR selectively affect amelanotic and melanotic melanoma cells vitality by decreasing the intracellular thiol level.

The study which characterised the anti-tumor and pro-apoptotic effect of MIM-1 on COLO829 cells also showed a significant increase in the cell amount with reduced thiols [5], which may indicate that its anti-cancer mechanism is by affecting the level of reduced thiols.

Oxidative damage and ROS play an important role in many human diseases including cancer. Cells constantly generate ROS—reactive oxygen species (highly reactive and unstable molecules) during aerobic metabolism. Oxidative stress occurs in cells when the generation of ROS overwhelms the cell’s antioxidant defenses. ROS causes lipid peroxidation and thus damages the cell membranes. ROS can induce tumor cell death initiating oxidative stress [47,48]. Tumor cells have a different redox balance and produce elevated levels of ROS compared to the normal cells and this identifies ROS manipulation as a potential target for cancer therapies. Anti-cancer signaling of ROS can be targeted as a new therapy, by the increased production of ROS levels to toxic levels and exhaustion of the antioxidant system capacity causing programmed cell death [49,50]. The fluorogenic dye H_2_DCFDA was used to detect ROS production [51].

The H_2_DCFDA assay revealed the increase in ROS level in melanotic melanoma cells exposed to KTP (in both concentration—0.5 mM and 1.0 mM) combined with UVAR by 25% and 440%, respectively, compared to the control (Figure 7).

In our study, it was demonstrated a loss of GSH in melanoma cells as an effect of treatment with fluoroquinolone antibiotic—lomefloxacin correlated well with the production of reactive oxygen species (ROS), indicating that the loss of GSH might result from excessive formation of oxidative stress [50]. Therefore, it may be concluded that the showed ability of ketoprofen applied in combination with UVAR to induce apoptosis via an intrinsic/mitochondrial signaling pathway may result from ROS overproduction.

## 4. Materials and Methods

### 4.1. Materials

Growth medium RPMI 1640, trypsin/EDTA and fetal bovine serum were purchased from Cytogen (Zgierz, Poland). A growth medium M-254 and human melanocytes growth suplement-2 (HMGS-2) were acquired from Cascade Biologics (Portland, OR, USA). NC-Slides (A2 and A8), Via-1-Cassette (containing acridine orange and DAPI), Solution 3 (1 μg/mL DAPI, 0.1% triton X-100), Solution 5 (400 μg/mL VitaBright-48, 500 μg/mL propidium iodide, 1.2 μg/mL acridine orange), Solution 7 (200 μg/mL JC-1), Solution 8 (1 μg/mL DAPI), Solution 15 (500 μg/mL Hoechst 33342), and Solution 16 (500 μg/mL propidium iodide) were obtained from ChemoMetec (Lillerød, Denmark). Annexin V-CF488A conjugate and Annexin V binding buffer were obtained from Biotium, Inc. (Fremont, CA, USA). Ketoprofen—KTP (chemical name: (RS)-2-(3-benzoylphenyl)-propionic acid), amphotericin B, penicillin, and dimethyl sulphoxide (DMSO) were obtained from Sigma-Aldrich Inc. (St. Louis, MO, USA). Other chemicals were from POCH S.A. (Gliwice, Poland).

### 4.2. Cell Lines and Cell Culture

COLO829 cells—the human melanotic melanoma cells—(American Type Culture Collection—ATCC, CRL-1974, Manassas, VA, USA) were cultivated in Roswell Park Memorial Institute (RPMI) 1640 medium supplemented with L-glutamine, heat-inactivated fetal bovine serum (10%). HEMn-DP—human epidermal melanocytes, neonatal, darkly pigmented were purchased from Cascade Biologics (Portland, OR, USA). The cells were maintained in M-254 medium with human melanocytes growth suplement-2. All media were complemented by antibiotics: neomycin (10 μg/mL), penicillin (100 U/mL), amphotericin B (0.25 μg/mL). The cells were maintained in an incubator that provides optimal growth conditions for the cell culture: 5% CO_2_, 37 °C. All experiments were performed on cells from passages 5 to 10.

Cells were irradiated by UVA (λ_max_ = 365 nm) using a filtered lamp BVL-8.LM (Vilber Lourmat, France) at dose of 2 J/cm^2^ (an intensity of 720 μW/cm^2^ for 46 min). Prior to irradiation, cells were pre-treated with KTP for 24 h. The drug solutions and culture medium were replaced with PBS solution, immediately before irradiation. Non-irradiated cells were placed in a CO_2_ incubator. After irradiation, the cells were post-incubated with the drug-free medium for 24 h.

### 4.3. Cells Treatment and Counting Assay

Melanoma cells (COLO829) and melanocytes (HEMn-DP) cells were seeded in Petri dish (100 mm × 20 mm)—1 × 10^6^ cells per dish, and incubated for 24 h. Then, the cells were pre-incubated with KTP solutions (0.5 mM and 1.0 mM) for 24 h. Afterwards, the cells were exposed to UVAR according to the procedure described above. Following post-incubation, cells were harvested and then counted using fluorescent imaging cytometer NucleoCounter NC-3000 (ChemoMetec, Lillerød, Denmark). In this method, non fixed cells are loaded into Via1-Cassettes (ChemoMetec, Lillerød, Denmark) and stained with acridine orange and DAPI in order to detect the total cell population or non-viable cells, respectively.

### 4.4. Detection of Phosphatidylserine Externalization in Apoptotic Cells

Staining cells with fluorescent conjugates of annexin V and propidium iodide (PI) is commonly used to identify apoptotic cells. COLO829 and HEMn-DP cells were seeded in Petri dish (100 mm × 20 mm)—1 × 10^6^ cells per dish, for 24 h. Subsequently, the cells were pre-treated with KTP (0.5 mM and 1.0 mM) for 24 h. Then, the cells were irradiated with UVAR, as described above. Cell suspensions were stained with Annexin V-CF488A conjugate and Hoechst 33342 (37 °C, 15 min). Stained cells were then centrifuged and washed with Annexin V binding buffer. Finally, the cell pellets were resuspended in Annexin V binding buffer supplemented with propidium iodide and loaded into NC-Slides A2. The analysis was performed using the NucleoCounter NC-3000.

### 4.5. Detection of Mitochondrial Depolarization

Mitochondrial membrane potential changes were evaluated by the use of the JC-1 (5,5′,6,6′-tetrachloro-1,1′,3,3′-tetraethylbenzimidazolocarbocyanine iodide) staining and image cytometry technique. Melanotic melanoma cells and melanocytes were treated with KTP at concentrations of 0.5 mM and 1.0 mM for 24 h. Subsequently, the cells were irradiated with UVAR. After 24 h post-incubation with culture medium (without drug), the cells were counted using the image cytometer. Cell suspensions (1 × 10^6^ cells/mL) were stained with Solution 7 (200 µg/mL JC-1) (20 min, 37 °C). Following washing with PBS, the cells were resuspended in Solution 8 (1 µg/mL DAPI in PBS) and analysed immediately.

### 4.6. Cell Cycle Analysis and DNA Fragmentation Assay

The cell cycle analysis of COLO829 melanoma cells and melanocytes was performed by the use of the fluorescence image cytometer NC-3000. In brief, the cells were pre-treated with the studied drug for 24 h. After the treatment, the cells were exposed to UVAR and post-incubated with drug-free medium for 24 h. Then, the cells were trypsinised, suspended in PBS (1 × 10^6^ cells/mL), and fixed with 70% cold ethanol. The samples were stained with Solution 3, loaded into NC-Slide A8, and analyzed cytometrically using the Fixed Cell Cycle-DAPI or DNA fragmentation assay protocol.

### 4.7. Assessment of Intracellular GSH Levels

GSH levels in COLO829 cells were assessed using the NC-3000 fluorescence image cytometer, as described previously [3]. In brief, cells were seeded in Petri dish (100 mm × 20 mm) − 1 × 10^6^ cells per dish and incubated in RPMI growth medium for 24 h. Then, the cells were pre-treated with KTP solutions (0.5 mM and 1.0 mM). Subsequently, the cells were exposed to UVAR. After completing the procedure the cells were detached and counted. Subsequently, 1 × 10^6^ cells were suspended in 0.5 mL of PBS, stained with Solution 5 (containing VitaBright-48, PI, AO) and analysed.

### 4.8. ROS Detection Assay—H_2_DCFDA

H_2_DCFDA reagent was used to assess the ROS generation in melanotic melanoma cells (COLO829) after combined treatment with KTP and UVAR. In brief, cells were placed in a 96-well dark microplate (2.5 × 10^3^ cells/well) in a growth medium and incubated for 24 h. After incubation, the medium was removed and the cells were treated with KTP (0.5 mM and 1.0 mM). After the treatment, the cells were exposed to UVAR and post-incubated with drug-free medium for 24 h. Cells were then treated with H_2_DCFDA for 30 min (in the dark) and washed twice with PBS. The fluorescence intensity (lex = 485 nm, lem = 530 nm) was measured using the microplate reader Infinite 200 Pro. The obtained results were normalised to a cell number and expressed as a percentage of control.

### 4.9. Statistical Data Analysis

GraphPad Prism 7 (GraphPad Software, San Diego, CA, USA) was used for the statistical analysis. All results are presented as mean (mean values of at least three separate experiments) ± SD. One-way ANOVA (the influence of UVAR or KTP) and two way ANOVA (the influence of UVAR and KTP) analysis of variance followed by Tukey’s test were applied to compare the means. Differences at of *p* < 0.05 (* *p* < 0.05; ** *p* < 0.001) were regarded as significant.

## 5. Conclusions

Melanoma remains a serious health problem, and the number of people suffering from this type of cancer increases every year. Unfortunately, none of the currently used strategies in the management of melanoma provides satisfactory results, thus examination of new drug with possible application in cancer therapy is desirable. In our study, we determined for the first time the antitumor effects of co-therapy with ketoprofen and UVA irradiation on melanotic melanoma cells and estimated its influence on normal cells—human melanocytes. The therapy combining the use of pre-incubation with KTP and UVAR causes a significant increase in the anti-proliferative properties of ketoprofen towards COLO829 melanoma cells. Moreover, the co-exposure of COLO829 cells to KTP and UVAR induced apoptosis shown as the mitochondrial membrane breakdown, cell-cycle deregulation, and DNA fragmentation. Finally, treatment of the studied melanoma cells with the combination of drug and UVAR led to GSH depletion showing its pro-apoptotic action based on ROS overproduction. Unlike to COLO829 melanoma cells, melanocytes subjected to the same procedures showed no significant changes in the polarization of the mitochondrial membrane, apoptosis, and DNA fragmentation induction.

In summary, the presented selective mode of action of the tested therapy strengthened by the anti-proliferative and pro-apoptotic effect of ketoprofen used in combination with UVAR towards melanotic melanoma cells may constitute a new insight into the possible application of the studied agents in the photochemotherapy of malignant melanoma.

## Data Availability

The data that support the findings of this study are available from the corresponding author upon reasonable request.

## Abbreviations

5-MOP	5-methoxypsoralen
8-MOP	8-methoxypsoralen
AO	acridine orange
COLO829	metastatic melanotic melanoma cell line
DMSO	dimethyl sulfoxide
GSH	reduced glutathione
HEMn-DP	human epidermal melanocytes, neonatal, darkly pigmented
HMGS-2	human melanocyte growth supplement-2
KTP	ketoprofen
PBS	phosphate buffered saline
PI	propidium iodide
PS	phosphatidylserine
PUVA	Psoralen Ultra-Violet A
ROS	reactive oxygen species
UVAR	ultraviolet A radiation
